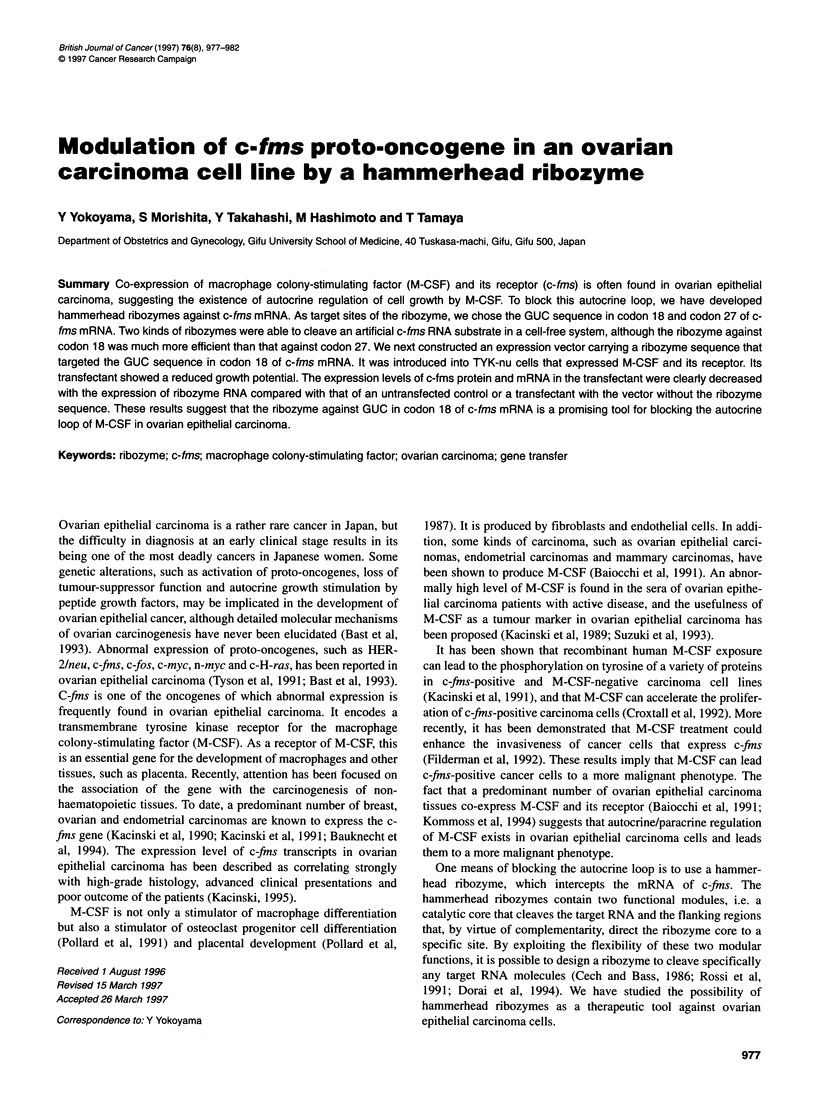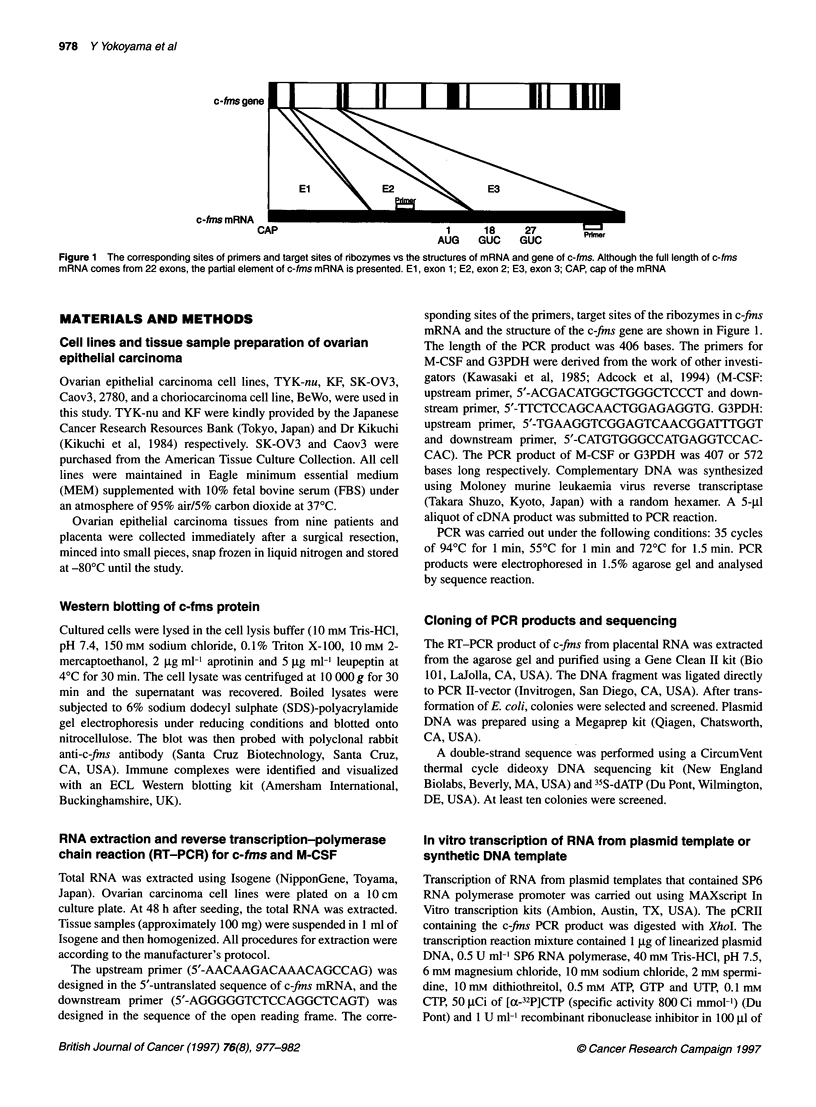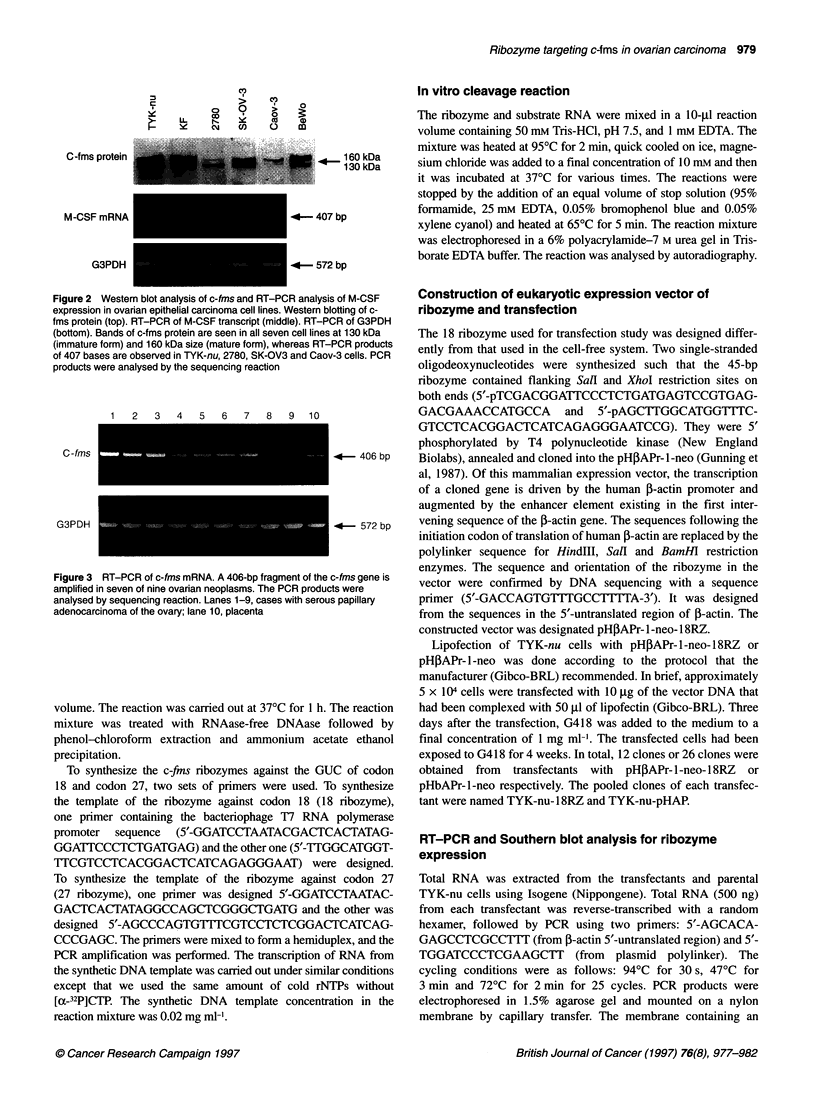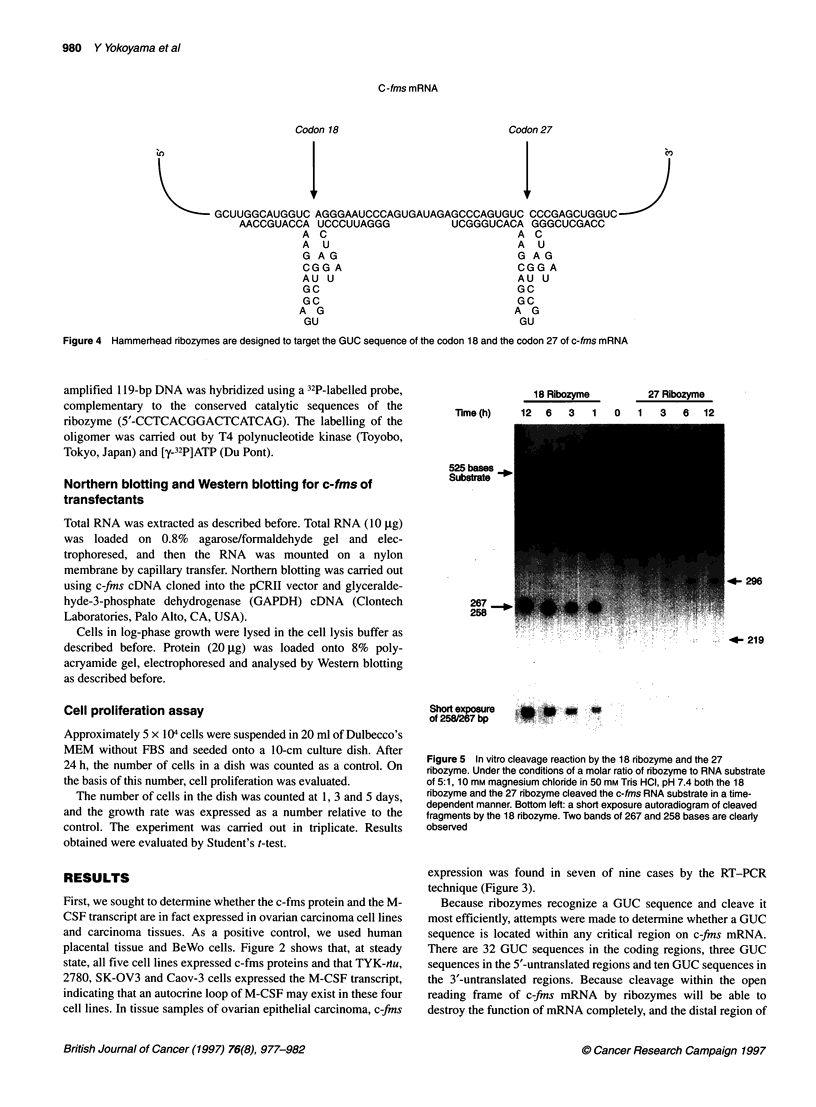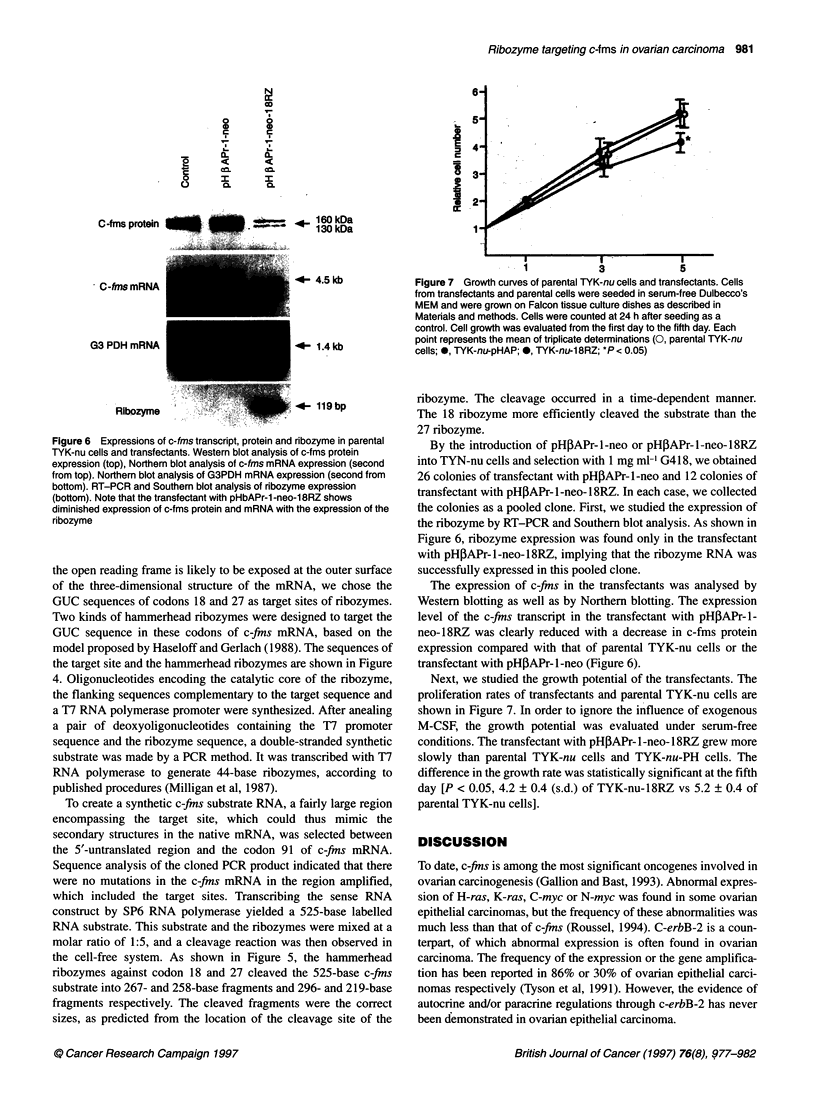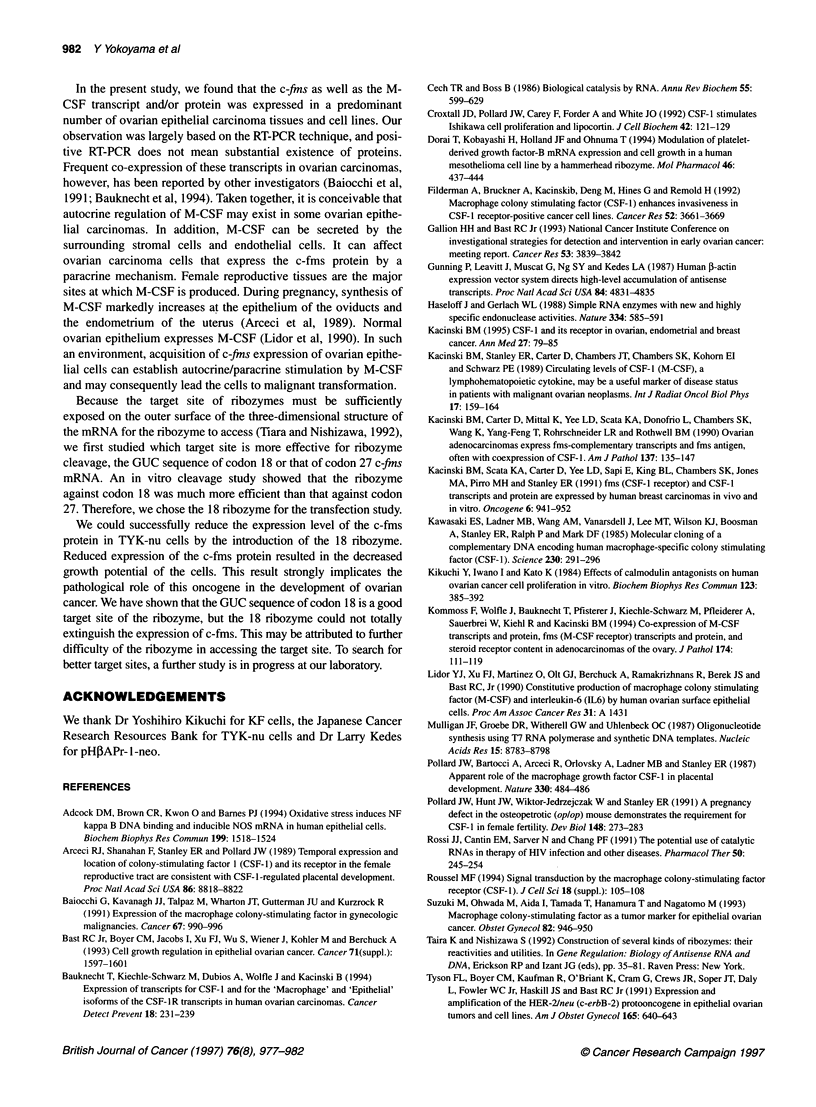# Modulation of c-fms proto-oncogene in an ovarian carcinoma cell line by a hammerhead ribozyme.

**DOI:** 10.1038/bjc.1997.496

**Published:** 1997

**Authors:** Y. Yokoyama, S. Morishita, Y. Takahashi, M. Hashimoto, T. Tamaya

**Affiliations:** Department of Obstetrics and Gynecology, Gifu University School of Medicine, Japan.

## Abstract

**Images:**


*British Journal of Cancer* (1997) **76**(8), 977–982
© 1997 Cancer Research Campaign

### Modulation of c-*fms* proto-oncogene in an ovarian carcinoma cell line by a hammerhead ribozyme

**Y Yokoyama, S Morishita, Y Takahashi, M Hashimoto and T Tamaya**

Department of Obstetrics and Gynecology, Gifu University School of Medicine, 40 Tuskasa-machi, Gifu, Gifu 500, Japan

**Summary** Co-expression of macrophage colony-stimulating factor (M-CSF) and its receptor (c-*fms*) is often found in ovarian epithelial carcinoma, suggesting the existence of autocrine regulation of cell growth by M-CSF. To block this autocrine loop, we have developed hammerhead ribozymes against c-*fms* mRNA. As target sites of the ribozyme, we chose the GUC sequence in codon 18 and codon 27 of c-*fms* mRNA. Two kinds of ribozymes were able to cleave an artificial c-*fms* RNA substrate in a cell-free system, although the ribozyme against codon 18 was much more efficient than that against codon 27. We next constructed an expression vector carrying a ribozyme sequence that targeted the GUC sequence in codon 18 of c-*fms* mRNA. It was introduced into TYK-nu cells that expressed M-CSF and its receptor. Its transfectant showed a reduced growth potential. The expression levels of c-fms protein and mRNA in the transfectant were clearly decreased with the expression of ribozyme RNA compared with that of an untransfected control or a transfectant with the vector without the ribozyme sequence. These results suggest that the ribozyme against GUC in codon 18 of c-*fms* mRNA is a promising tool for blocking the autocrine loop of M-CSF in ovarian epithelial carcinoma.

**Keywords:** ribozyme; c-*fms*; macrophage colony-stimulating factor; ovarian carcinoma; gene transfer

Ovarian epithelial carcinoma is a rather rare cancer in Japan, but the difficulty in diagnosis at an early clinical stage results in its being one of the most deadly cancers in Japanese women. Some genetic alterations, such as activation of proto-oncogenes, loss of tumour-suppressor function and autocrine growth stimulation by peptide growth factors, may be implicated in the development of ovarian epithelial cancer, although detailed molecular mechanisms of ovarian carcinogenesis have never been elucidated (Bast et al, 1993). Abnormal expression of proto-oncogenes, such as HER-2/*neu*, c-*fms*, c-*fos*, c-*myc*, n-*myc* and c-H-*ras*, has been reported in ovarian epithelial carcinoma (Tyson et al, 1991; Bast et al, 1993). C-*fms* is one of the oncogenes of which abnormal expression is frequently found in ovarian epithelial carcinoma. It encodes a transmembrane tyrosine kinase receptor for the macrophage colony-stimulating factor (M-CSF). As a receptor of M-CSF, this is an essential gene for the development of macrophages and other tissues, such as placenta. Recently, attention has been focused on the association of the gene with the carcinogenesis of non-haematopoietic tissues. To date, a predominant number of breast, ovarian and endometrial carcinomas are known to express the c-*fms* gene (Kacinski et al, 1990; Kacinski et al, 1991; Bauknecht et al, 1994). The expression level of c-*fms* transcripts in ovarian epithelial carcinoma has been described as correlating strongly with high-grade histology, advanced clinical presentations and poor outcome of the patients (Kacinski, 1995).

M-CSF is not only a stimulator of macrophage differentiation but also a stimulator of osteoclast progenitor cell differentiation (Pollard et al, 1991) and placental development (Pollard et al, 1987). It is produced by fibroblasts and endothelial cells. In addition, some kinds of carcinoma, such as ovarian epithelial carcinomas, endometrial carcinomas and mammary carcinomas, have been shown to produce M-CSF (Baiocchi et al, 1991). An abnormally high level of M-CSF is found in the sera of ovarian epithelial carcinoma patients with active disease, and the usefulness of M-CSF as a tumour marker in ovarian epithelial carcinoma has been proposed (Kacinski et al, 1989; Suzuki et al, 1993).

It has been shown that recombinant human M-CSF exposure can lead to the phosphorylation on tyrosine of a variety of proteins in c-*fms*-positive and M-CSF-negative carcinoma cell lines (Kacinski et al, 1991), and that M-CSF can accelerate the proliferation of c-*fms*-positive carcinoma cells (Croxtall et al, 1992). More recently, it has been demonstrated that M-CSF treatment could enhance the invasiveness of cancer cells that express c-*fms* (Filderman et al, 1992). These results imply that M-CSF can lead c-*fms*-positive cancer cells to a more malignant phenotype. The fact that a predominant number of ovarian epithelial carcinoma tissues co-express M-CSF and its receptor (Baiocchi et al, 1991; Kommoss et al, 1994) suggests that autocrine/paracrine regulation of M-CSF exists in ovarian epithelial carcinoma cells and leads them to a more malignant phenotype.

One means of blocking the autocrine loop is to use a hammerhead ribozyme, which intercepts the mRNA of c-*fms*. The hammerhead ribozymes contain two functional modules, i.e. a catalytic core that cleaves the target RNA and the flanking regions that, by virtue of complementarity, direct the ribozyme core to a specific site. By exploiting the flexibility of these two modular functions, it is possible to design a ribozyme to cleave specifically any target RNA molecules (Cech and Bass, 1986; Rossi et al, 1991; Dorai et al, 1994). We have studied the possibility of hammerhead ribozymes as a therapeutic tool against ovarian epithelial carcinoma cells.

*Received 1 August 1996*
*Revised 15 March 1997*
*Accepted 26 March 1997*

*Correspondence to:* Y Yokoyama

**Figure 1** The corresponding sites of primers and target sites of ribozymes vs the structures of mRNA and gene of c-*fms*. Although the full length of c-*fms* mRNA comes from 22 exons, the partial element of c-*fms* mRNA is presented. E1, exon 1; E2, exon 2; E3, exon 3; CAP, cap of the mRNA

### MATERIALS AND METHODS

### Cell lines and tissue sample preparation of ovarian epithelial carcinoma

Ovarian epithelial carcinoma cell lines, TYK-*nu*, KF, SK-OV3, Caov3, 2780, and a choriocarcinoma cell line, BeWo, were used in this study. TYK-nu and KF were kindly provided by the Japanese Cancer Research Resources Bank (Tokyo, Japan) and Dr Kikuchi (Kikuchi et al, 1984) respectively. SK-OV3 and Caov3 were purchased from the American Tissue Culture Collection. All cell lines were maintained in Eagle minimum essential medium (MEM) supplemented with 10% fetal bovine serum (FBS) under an atmosphere of 95% air/5% carbon dioxide at 37°C.

Ovarian epithelial carcinoma tissues from nine patients and placenta were collected immediately after a surgical resection, minced into small pieces, snap frozen in liquid nitrogen and stored at –80°C until the study.

### Western blotting of c-fms protein

Cultured cells were lysed in the cell lysis buffer (10 mM Tris-HCl, pH 7.4, 150 mM sodium chloride, 0.1% Triton X-100, 10 mM 2-mercaptoethanol, 2 μg ml$^{-1}$ aprotinin and 5 μg ml$^{-1}$ leupeptin at 4°C for 30 min. The cell lysate was centrifuged at 10 000 ***g*** for 30 min and the supernatant was recovered. Boiled lysates were subjected to 6% sodium dodecyl sulphate (SDS)-polyacrylamide gel electrophoresis under reducing conditions and blotted onto nitrocellulose. The blot was then probed with polyclonal rabbit anti-c-*fms* antibody (Santa Cruz Biotechnology, Santa Cruz, CA, USA). Immune complexes were identified and visualized with an ECL Western blotting kit (Amersham International, Buckinghamshire, UK).

### RNA extraction and reverse transcription–polymerase chain reaction (RT–PCR) for c-*fms* and M-CSF

Total RNA was extracted using Isogene (NipponGene, Toyama, Japan). Ovarian carcinoma cell lines were plated on a 10 cm culture plate. At 48 h after seeding, the total RNA was extracted. Tissue samples (approximately 100 mg) were suspended in 1 ml of Isogene and then homogenized. All procedures for extraction were according to the manufacturer's protocol.

The upstream primer (5′-AACAAGACAAACAGCCAG) was designed in the 5′-untranslated sequence of c-*fms* mRNA, and the downstream primer (5′-AGGGGGTCTCCAGGCTCAGT) was designed in the sequence of the open reading frame. The corresponding sites of the primers, target sites of the ribozymes in c-*fms* mRNA and the structure of the c-*fms* gene are shown in Figure 1. The length of the PCR product was 406 bases. The primers for M-CSF and G3PDH were derived from the work of other investigators (Kawasaki et al, 1985; Adcock et al, 1994) (M-CSF: upstream primer, 5′-ACGACATGGCTGGGCTCCCT and downstream primer, 5′-TTCTCCAGCAACTGGAGAGGTG. G3PDH: upstream primer, 5′-TGAAGGTCGGAGTCAACGGATTTGGT and downstream primer, 5′-CATGTGGGCCATGAGGTCCACCAC). The PCR product of M-CSF or G3PDH was 407 or 572 bases long respectively. Complementary DNA was synthesized using Moloney murine leukaemia virus reverse transcriptase (Takara Shuzo, Kyoto, Japan) with a random hexamer. A 5-μl aliquot of cDNA product was submitted to PCR reaction.

PCR was carried out under the following conditions: 35 cycles of 94°C for 1 min, 55°C for 1 min and 72°C for 1.5 min. PCR products were electrophoresed in 1.5% agarose gel and analysed by sequence reaction.

### Cloning of PCR products and sequencing

The RT–PCR product of c-*fms* from placental RNA was extracted from the agarose gel and purified using a Gene Clean II kit (Bio 101, LaJolla, CA, USA). The DNA fragment was ligated directly to PCR II-vector (Invitrogen, San Diego, CA, USA). After transformation of *E. coli*, colonies were selected and screened. Plasmid DNA was prepared using a Megaprep kit (Qiagen, Chatsworth, CA, USA).

A double-strand sequence was performed using a CircumVent thermal cycle dideoxy DNA sequencing kit (New England Biolabs, Beverly, MA, USA) and $^{35}$S-dATP (Du Pont, Wilmington, DE, USA). At least ten colonies were screened.

### In vitro transcription of RNA from plasmid template or synthetic DNA template

Transcription of RNA from plasmid templates that contained SP6 RNA polymerase promoter was carried out using MAXscript In Vitro transcription kits (Ambion, Austin, TX, USA). The pCRII containing the c-*fms* PCR product was digested with *Xho*I. The transcription reaction mixture contained 1 μg of linearized plasmid DNA, 0.5 U ml$^{-1}$ SP6 RNA polymerase, 40 mM Tris-HCl, pH 7.5, 6 mM magnesium chloride, 10 mM sodium chloride, 2 mM spermidine, 10 mM dithiothreitol, 0.5 mM ATP, GTP and UTP, 0.1 mM CTP, 50 μCi of [α-$^{32}$P]CTP (specific activity 800 Ci mmol$^{-1}$) (Du Pont) and 1 U ml$^{-1}$ recombinant ribonuclease inhibitor in 100 μl of

© Cancer Research Campaign 1997

**Figure 2** Western blot analysis of c-*fms* and RT–PCR analysis of M-CSF expression in ovarian epithelial carcinoma cell lines. Western blotting of c-fms protein (top). RT–PCR of M-CSF transcript (middle). RT–PCR of G3PDH (bottom). Bands of c-fms protein are seen in all seven cell lines at 130 kDa (immature form) and 160 kDa size (mature form), whereas RT–PCR products of 407 bases are observed in TYK-*nu*, 2780, SK-OV3 and Caov-3 cells. PCR products were analysed by the sequencing reaction

**Figure 3** RT–PCR of c-*fms* mRNA. A 406-bp fragment of the c-*fms* gene is amplified in seven of nine ovarian neoplasms. The PCR products were analysed by sequencing reaction. Lanes 1–9, cases with serous papillary adenocarcinoma of the ovary; lane 10, placenta volume. The reaction was carried out at 37°C for 1 h. The reaction mixture was treated with RNAase-free DNAase followed by phenol–chloroform extraction and ammonium acetate ethanol precipitation.

To synthesize the c-*fms* ribozymes against the GUC of codon 18 and codon 27, two sets of primers were used. To synthesize the template of the ribozyme against codon 18 (18 ribozyme), one primer containing the bacteriophage T7 RNA polymerase promoter sequence (5′-GGATCCTAATACGACTCACTATAG-GGATTCCCTCTGATGAG) and the other one (5′-TTGGCATGGT-TTCGTCCTCACGGACTCATCAGAGGGAAT) were designed. To synthesize the template of the ribozyme against codon 27 (27 ribozyme), one primer was designed 5′-GGATCCTAATAC-GACTCACTATAGGCCAGCTCGGGCTGATG and the other was designed 5′-AGCCCAGTGTTTCGTCCTCTCGGACTCATCAG-CCCGAGC. The primers were mixed to form a hemiduplex, and the PCR amplification was performed. The transcription of RNA from the synthetic DNA template was carried out under similar conditions except that we used the same amount of cold rNTPs without [$\alpha$-$^{32}$P]CTP. The synthetic DNA template concentration in the reaction mixture was 0.02 mg ml$^{-1}$.

### In vitro cleavage reaction

The ribozyme and substrate RNA were mixed in a 10-µl reaction volume containing 50 mM Tris-HCl, pH 7.5, and 1 mM EDTA. The mixture was heated at 95°C for 2 min, quick cooled on ice, magnesium chloride was added to a final concentration of 10 mM and then it was incubated at 37°C for various times. The reactions were stopped by the addition of an equal volume of stop solution (95% formamide, 25 mM EDTA, 0.05% bromophenol blue and 0.05% xylene cyanol) and heated at 65°C for 5 min. The reaction mixture was electrophoresed in a 6% polyacrylamide–7 M urea gel in Tris-borate EDTA buffer. The reaction was analysed by autoradiography.

### Construction of eukaryotic expression vector of ribozyme and transfection

The 18 ribozyme used for transfection study was designed differently from that used in the cell-free system. Two single-stranded oligodeoxynucleotides were synthesized such that the 45-bp ribozyme contained flanking *Sal*I and *Xho*I restriction sites on both ends (5′-pTCGACGGATTCCCTCTGATGAGTCCGTGAG-GACGAAACCATGCCA and 5′-pAGCTTGGCATGGTTTC-GTCCTCACGGACTCATCAGAGGGAATCCG). They were 5′ phosphorylated by T4 polynucleotide kinase (New England Biolabs), annealed and cloned into the pHβAPr-1-neo (Gunning et al, 1987). Of this mammalian expression vector, the transcription of a cloned gene is driven by the human β-actin promoter and augmented by the enhancer element existing in the first intervening sequence of the β-actin gene. The sequences following the initiation codon of translation of human β-actin are replaced by the polylinker sequence for *Hin*dIII, *Sal*I and *BamH*I restriction enzymes. The sequence and orientation of the ribozyme in the vector were confirmed by DNA sequencing with a sequence primer (5′-GACCAGTGTTTGCCTTTTA-3′). It was designed from the sequences in the 5′-untranslated region of β-actin. The constructed vector was designated pHβAPr-1-neo-18RZ.

Lipofection of TYK-*nu* cells with pHβAPr-1-neo-18RZ or pHβAPr-1-neo was done according to the protocol that the manufacturer (Gibco-BRL) recommended. In brief, approximately $5 \times 10^4$ cells were transfected with 10 µg of the vector DNA that had been complexed with 50 µl of lipofectin (Gibco-BRL). Three days after the transfection, G418 was added to the medium to a final concentration of 1 mg ml$^{-1}$. The transfected cells had been exposed to G418 for 4 weeks. In total, 12 clones or 26 clones were obtained from transfectants with pHβAPr-1-neo-18RZ or pHbAPr-1-neo respectively. The pooled clones of each transfectant were named TYK-nu-18RZ and TYK-nu-pHAP.

### RT–PCR and Southern blot analysis for ribozyme expression

Total RNA was extracted from the transfectants and parental TYK-nu cells using Isogene (Nippongene). Total RNA (500 ng) from each transfectant was reverse-transcribed with a random hexamer, followed by PCR using two primers: 5′-AGCACA-GAGCCTCGCCTTT (from β-actin 5′-untranslated region) and 5′-TGGATCCCTCGAAGCTT (from plasmid polylinker). The cycling conditions were as follows: 94°C for 30 s, 47°C for 3 min and 72°C for 2 min for 25 cycles. PCR products were electrophoresed in 1.5% agarose gel and mounted on a nylon membrane by capillary transfer. The membrane containing an

**Figure 4** Hammerhead ribozymes are designed to target the GUC sequence of the codon 18 and the codon 27 of c-*fms* mRNA

amplified 119-bp DNA was hybridized using a $^{32}$P-labelled probe, complementary to the conserved catalytic sequences of the ribozyme (5′-CCTCACGGACTCATCAG). The labelling of the oligomer was carried out by T4 polynucleotide kinase (Toyobo, Tokyo, Japan) and [γ-$^{32}$P]ATP (Du Pont).

### Northern blotting and Western blotting for c-*fms* of transfectants

Total RNA was extracted as described before. Total RNA (10 μg) was loaded on 0.8% agarose/formaldehyde gel and electrophoresed, and then the RNA was mounted on a nylon membrane by capillary transfer. Northern blotting was carried out using c-*fms* cDNA cloned into the pCRII vector and glyceraldehyde-3-phosphate dehydrogenase (GAPDH) cDNA (Clontech Laboratories, Palo Alto, CA, USA).

Cells in log-phase growth were lysed in the cell lysis buffer as described before. Protein (20 μg) was loaded onto 8% polyacryamide gel, electrophoresed and analysed by Western blotting as described before.

### Cell proliferation assay

Approximately 5 × 10$^4$ cells were suspended in 20 ml of Dulbecco's MEM without FBS and seeded onto a 10-cm culture dish. After 24 h, the number of cells in a dish was counted as a control. On the basis of this number, cell proliferation was evaluated.

The number of cells in the dish was counted at 1, 3 and 5 days, and the growth rate was expressed as a number relative to the control. The experiment was carried out in triplicate. Results obtained were evaluated by Student's *t*-test.

### RESULTS

First, we sought to determine whether the c-fms protein and the M-CSF transcript are in fact expressed in ovarian carcinoma cell lines and carcinoma tissues. As a positive control, we used human placental tissue and BeWo cells. Figure 2 shows that, at steady state, all five cell lines expressed c-fms proteins and that TYK-*nu*, 2780, SK-OV3 and Caov-3 cells expressed the M-CSF transcript, indicating that an autocrine loop of M-CSF may exist in these four cell lines. In tissue samples of ovarian epithelial carcinoma, c-*fms* expression was found in seven of nine cases by the RT–PCR technique (Figure 3).

**Figure 5** In vitro cleavage reaction by the 18 ribozyme and the 27 ribozyme. Under the conditions of a molar ratio of ribozyme to RNA substrate of 5:1, 10 mM magnesium chloride in 50 mM Tris HCl, pH 7.4 both the 18 ribozyme and the 27 ribozyme cleaved the c-*fms* RNA substrate in a time-dependent manner. Bottom left: a short exposure autoradiogram of cleaved fragments by the 18 ribozyme. Two bands of 267 and 258 bases are clearly observed

Because ribozymes recognize a GUC sequence and cleave it most efficiently, attempts were made to determine whether a GUC sequence is located within any critical region on c-*fms* mRNA. There are 32 GUC sequences in the coding regions, three GUC sequences in the 5′-untranslated regions and ten GUC sequences in the 3′-untranslated regions. Because cleavage within the open reading frame of c-*fms* mRNA by ribozymes will be able to destroy the function of mRNA completely, and the distal region of

*© Cancer Research Campaign 1997*

**Figure 6** Expressions of c-*fms* transcript, protein and ribozyme in parental TYK-nu cells and transfectants. Western blot analysis of c-fms protein expression (top), Northern blot analysis of c-*fms* mRNA expression (second from top). Northern blot analysis of G3PDH mRNA expression (second from bottom). RT–PCR and Southern blot analysis of ribozyme expression (bottom). Note that the transfectant with pHbAPr-1-neo-18RZ shows diminished expression of c-fms protein and mRNA with the expression of the ribozyme

**Figure 7** Growth curves of parental TYK-*nu* cells and transfectants. Cells from transfectants and parental cells were seeded in serum-free Dulbecco's MEM and were grown on Falcon tissue culture dishes as described in Materials and methods. Cells were counted at 24 h after seeding as a control. Cell growth was evaluated from the first day to the fifth day. Each point represents the mean of triplicate determinations (○, parental TYK-*nu* cells; ●, TYK-*nu*-pHAP; ●, TYK-*nu*-18RZ; *$P < 0.05$)

the open reading frame is likely to be exposed at the outer surface of the three-dimensional structure of the mRNA, we chose the GUC sequences of codons 18 and 27 as target sites of ribozymes. Two kinds of hammerhead ribozymes were designed to target the GUC sequence in these codons of c-*fms* mRNA, based on the model proposed by Haseloff and Gerlach (1988). The sequences of the target site and the hammerhead ribozymes are shown in Figure 4. Oligonucleotides encoding the catalytic core of the ribozyme, the flanking sequences complementary to the target sequence and a T7 RNA polymerase promoter were synthesized. After anealing a pair of deoxyoligonucleotides containing the T7 promoter sequence and the ribozyme sequence, a double-stranded synthetic substrate was made by a PCR method. It was transcribed with T7 RNA polymerase to generate 44-base ribozymes, according to published procedures (Milligan et al, 1987).

To create a synthetic c-*fms* substrate RNA, a fairly large region encompassing the target site, which could thus mimic the secondary structures in the native mRNA, was selected between the 5′-untranslated region and the codon 91 of c-*fms* mRNA. Sequence analysis of the cloned PCR product indicated that there were no mutations in the c-*fms* mRNA in the region amplified, which included the target sites. Transcribing the sense RNA construct by SP6 RNA polymerase yielded a 525-base labelled RNA substrate. This substrate and the ribozymes were mixed at a molar ratio of 1:5, and a cleavage reaction was then observed in the cell-free system. As shown in Figure 5, the hammerhead ribozymes against codon 18 and 27 cleaved the 525-base c-*fms* substrate into 267- and 258-base fragments and 296- and 219-base fragments respectively. The cleaved fragments were the correct sizes, as predicted from the location of the cleavage site of the ribozyme. The cleavage occurred in a time-dependent manner. The 18 ribozyme more efficiently cleaved the substrate than the 27 ribozyme.

By the introduction of pHβAPr-1-neo or pHβAPr-1-neo-18RZ into TYN-nu cells and selection with 1 mg $ml^{-1}$ G418, we obtained 26 colonies of transfectant with pHβAPr-1-neo and 12 colonies of transfectant with pHβAPr-1-neo-18RZ. In each case, we collected the colonies as a pooled clone. First, we studied the expression of the ribozyme by RT–PCR and Southern blot analysis. As shown in Figure 6, ribozyme expression was found only in the transfectant with pHβAPr-1-neo-18RZ, implying that the ribozyme RNA was successfully expressed in this pooled clone.

The expression of c-*fms* in the transfectants was analysed by Western blotting as well as by Northern blotting. The expression level of the c-*fms* transcript in the transfectant with pHβAPr-1-neo-18RZ was clearly reduced with a decrease in c-fms protein expression compared with that of parental TYK-nu cells or the transfectant with pHβAPr-1-neo (Figure 6).

Next, we studied the growth potential of the transfectants. The proliferation rates of transfectants and parental TYK-nu cells are shown in Figure 7. In order to ignore the influence of exogenous M-CSF, the growth potential was evaluated under serum-free conditions. The transfectant with pHβAPr-1-neo-18RZ grew more slowly than parental TYK-*nu* cells and TYK-*nu*-PH cells. The difference in the growth rate was statistically significant at the fifth day [$P < 0.05$, 4.2 ± 0.4 (s.d.) of TYK-nu-18RZ vs 5.2 ± 0.4 of parental TYK-nu cells].

### DISCUSSION

To date, c-*fms* is among the most significant oncogenes involved in ovarian carcinogenesis (Gallion and Bast, 1993). Abnormal expression of H-*ras*, K-*ras*, C-*myc* or N-*myc* was found in some ovarian epithelial carcinomas, but the frequency of these abnormalities was much less than that of c-*fms* (Roussel, 1994). C-*erb*B-2 is a counterpart, of which abnormal expression is often found in ovarian carcinoma. The frequency of the expression or the gene amplification has been reported in 86% or 30% of ovarian epithelial carcinomas respectively (Tyson et al, 1991). However, the evidence of autocrine and/or paracrine regulations through c-*erb*B-2 has never been demonstrated in ovarian epithelial carcinoma.

In the present study, we found that the c-*fms* as well as the M-CSF transcript and/or protein was expressed in a predominant number of ovarian epithelial carcinoma tissues and cell lines. Our observation was largely based on the RT-PCR technique, and positive RT-PCR does not mean substantial existence of proteins. Frequent co-expression of these transcripts in ovarian carcinomas, however, has been reported by other investigators (Baiocchi et al, 1991; Bauknecht et al, 1994). Taken together, it is conceivable that autocrine regulation of M-CSF may exist in some ovarian epithelial carcinomas. In addition, M-CSF can be secreted by the surrounding stromal cells and endothelial cells. It can affect ovarian carcinoma cells that express the c-fms protein by a paracrine mechanism. Female reproductive tissues are the major sites at which M-CSF is produced. During pregnancy, synthesis of M-CSF markedly increases at the epithelium of the oviducts and the endometrium of the uterus (Arceci et al, 1989). Normal ovarian epithelium expresses M-CSF (Lidor et al, 1990). In such an environment, acquisition of c-*fms* expression of ovarian epithelial cells can establish autocrine/paracrine stimulation by M-CSF and may consequently lead the cells to malignant transformation.

Because the target site of ribozymes must be sufficiently exposed on the outer surface of the three-dimensional structure of the mRNA for the ribozyme to access (Tiara and Nishizawa, 1992), we first studied which target site is more effective for ribozyme cleavage, the GUC sequence of codon 18 or that of codon 27 c-*fms* mRNA. An in vitro cleavage study showed that the ribozyme against codon 18 was much more efficient than that against codon 27. Therefore, we chose the 18 ribozyme for the transfection study.

We could successfully reduce the expression level of the c-fms protein in TYK-nu cells by the introduction of the 18 ribozyme. Reduced expression of the c-fms protein resulted in the decreased growth potential of the cells. This result strongly implicates the pathological role of this oncogene in the development of ovarian cancer. We have shown that the GUC sequence of codon 18 is a good target site of the ribozyme, but the 18 ribozyme could not totally extinguish the expression of c-fms. This may be attributed to further difficulty of the ribozyme in accessing the target site. To search for better target sites, a further study is in progress at our laboratory.

### ACKNOWLEDGEMENTS

We thank Dr Yoshihiro Kikuchi for KF cells, the Japanese Cancer Research Resources Bank for TYK-nu cells and Dr Larry Kedes for pHβAPr-1-neo.

### REFERENCES

Adcock DM, Brown CR, Kwon O and Barnes PJ (1994) Oxidative stress induces NF kappa B DNA binding and inducible NOS mRNA in human epithelial cells. *Biochem Biophys Res Commun* **199**: 1518–1524

Arceci RJ, Shanahan F, Stanley ER and Pollard JW (1989) Temporal expression and location of colony-stimulating factor 1 (CSF-1) and its receptor in the female reproductive tract are consistent with CSF-1-regulated placental development. *Proc Natl Acad Sci USA* **86**: 8818–8822

Baiocchi G, Kavanagh JJ, Talpaz M, Wharton JT, Gutterman JU and Kurzrock R (1991) Expression of the macrophage colony-stimulating factor in gynecologic malignancies. *Cancer* **67**: 990–996

Bast RC Jr, Boyer CM, Jacobs I, Xu FJ, Wu S, Wiener J, Kohler M and Berchuck A (1993) Cell growth regulation in epithelial ovarian cancer. *Cancer* **71**(suppl.): 1597–1601

Bauknecht T, Kiechle-Schwarz M, Dubios A, Wolfle J and Kacinski B (1994) Expression of transcripts for CSF-1 and for the 'Macrophage' and 'Epithelial' isoforms of the CSF-1R transcripts in human ovarian carcinomas. *Cancer Detect Prevent* **18**: 231–239

Cech TR and Boss B (1986) Biological catalysis by RNA. *Annu Rev Biochem* **55**: 599–629

Croxtall JD, Pollard JW, Carey F, Forder A and White JO (1992) CSF-1 stimulates Ishikawa cell proliferation and lipocortin. *J Cell Biochem* **42**: 121–129

Dorai T, Kobayashi H, Holland JF and Ohnuma T (1994) Modulation of platelet-derived growth factor-B mRNA expression and cell growth in a human mesothelioma cell line by a hammerhead ribozyme. *Mol Pharmacol* **46**: 437–444

Filderman A, Bruckner A, Kacinskib, Deng M, Hines G and Remold H (1992) Macrophage colony stimulating factor (CSF-1) enhances invasiveness in CSF-1 receptor-positive cancer cell lines. *Cancer Res* **52**: 3661–3669

Gallion HH and Bast RC Jr (1993) National Cancer Institute Conference on investigational strategies for detection and intervention in early ovarian cancer: meeting report. *Cancer Res* **53**: 3839–3842

Gunning P, Leavitt J, Muscat G, Ng SY and Kedes LA (1987) Human β-actin expression vector system directs high-level accumulation of antisense transcripts. *Proc Natl Acad Sci USA* **84**: 4831–4835

Haseloff J and Gerlach WL (1988) Simple RNA enzymes with new and highly specific endonuclease activities. *Nature* **334**: 585–591

Kacinski BM (1995) CSF-1 and its receptor in ovarian, endometrial and breast cancer. *Ann Med* **27**: 79–85

Kacinski BM, Stanley ER, Carter D, Chambers JT, Chambers SK, Kohorn EI and Schwarz PE (1989) Circulating levels of CSF-1 (M-CSF), a lymphohematopoietic cytokine, may be a useful marker of disease status in patients with malignant ovarian neoplasms. *Int J Radiat Oncol Biol Phys* **17**: 159–164

Kacinski BM, Carter D, Mittal K, Yee LD, Scata KA, Donofrio L, Chambers SK, Wang K, Yang-Feng T, Rohrschneider LR and Rothwell BM (1990) Ovarian adenocarcinomas express fms-complementary transcripts and fms antigen, often with coexpression of CSF-1. *Am J Pathol* **137**: 135–147

Kacinski BM, Scata KA, Carter D, Yee LD, Sapi E, King BL, Chambers SK, Jones MA, Pirro MH and Stanley ER (1991) fms (CSF-1 receptor) and CSF-1 transcripts and protein are expressed by human breast carcinomas in vivo and in vitro. *Oncogene* **6**: 941–952

Kawasaki ES, Ladner MB, Wang AM, Vanarsdell J, Lee MT, Wilson KJ, Boosman A, Stanley ER, Ralph P and Mark DF (1985) Molecular cloning of a complementary DNA encoding human macrophage-specific colony stimulating factor (CSF-1). *Science* **230**: 291–296

Kikuchi Y, Iwano I and Kato K (1984) Effects of calmodulin antagonists on human ovarian cancer cell proliferation in vitro. *Biochem Biophys Res Commun* **123**: 385–392

Kommoss F, Wolfle J, Bauknecht T, Pfisterer J, Kiechle-Schwarz M, Pfleiderer A, Sauerbrei W, Kiehl R and Kacinski BM (1994) Co-expression of M-CSF transcripts and protein, fms (M-CSF receptor) transcripts and protein, and steroid receptor content in adenocarcinomas of the ovary. *J Pathol* **174**: 111–119

Lidor YJ, Xu FJ, Martinez O, Olt GJ, Berchuck A, Ramakrizhnans R, Berek JS and Bast RC, Jr (1990) Constitutive production of macrophage colony stimulating factor (M-CSF) and interleukin-6 (IL6) by human ovarian surface epithelial cells. *Proc Am Assoc Cancer Res* **31**: A 1431

Mulligan JF, Groebe DR, Witherell GW and Uhlenbeck OC (1987) Oligonucleotide synthesis using T7 RNA polymerase and synthetic DNA templates. *Nucleic Acids Res* **15**: 8783–8798

Pollard JW, Bartocci A, Arceci R, Orlovsky A, Ladner MB and Stanley ER (1987) Apparent role of the macrophage growth factor CSF-1 in placental development. *Nature* **330**: 484–486

Pollard JW, Hunt JW, Wiktor-Jedrzejczak W and Stanley ER (1991) A pregnancy defect in the osteopetrotic (*op/op*) mouse demonstrates the requirement for CSF-1 in female fertility. *Dev Biol* **148**: 273–283

Rossi JJ, Cantin EM, Sarver N and Chang PF (1991) The potential use of catalytic RNAs in therapy of HIV infection and other diseases. *Pharmacol Ther* **50**: 245–254

Roussel MF (1994) Signal transduction by the macrophage colony-stimulating factor receptor (CSF-1). *J Cell Sci* **18** (suppl.): 105–108

Suzuki M, Ohwada M, Aida I, Tamada T, Hanamura T and Nagatomo M (1993) Macrophage colony-stimulating factor as a tumor marker for epithelial ovarian cancer. *Obstet Gynecol* **82**: 946–950

Taira K and Nishizawa S (1992) Construction of several kinds of ribozymes: their reactivities and utilities. In *Gene Regulation: Biology of Antisense RNA and DNA*, Erickson RP and Izant JG (eds), pp. 35–81. Raven Press: New York.

Tyson FL, Boyer CM, Kaufman R, O'Briant K, Cram G, Crews JR, Soper JT, Daly L, Fowler WC Jr, Haskill JS and Bast RC Jr (1991) Expression and amplification of the HER-2/*neu* (c-*erb*B-2) protooncogene in epithelial ovarian tumors and cell lines. *Am J Obstet Gynecol* **165**: 640–643

 © Cancer Research Campaign 1997